# Drug delivery strategy in hepatocellular carcinoma therapy

**DOI:** 10.1186/s12964-021-00796-x

**Published:** 2022-03-05

**Authors:** Sisi Yang, Chengwei Cai, Huanqiu Wang, Xueqing Ma, Anwen Shao, Jifang Sheng, Chengbo Yu

**Affiliations:** 1 State Key Laboratory for Diagnosis and Treatment of Infectious Diseases, National Clinical Research Center for Infectious Diseases, Collaborative Innovation Center for Diagnosis and Treatment of Infectious Diseases, The First Affiliated Hospital, Zhejiang University School of Medicine, Hangzhou, Zhejiang Province China; 2 Department of Neurosurgery, The Second Affiliated Hospital of Zhejiang University School of Medicine, Hangzhou, Zhejiang Province China

**Keywords:** Hepatocellular carcinoma, Nanoparticle, Drug delivery, Tumor microenvironment

## Abstract

Hepatocellular carcinoma (HCC) is one of the most common malignant tumors worldwide, with high rates of recurrence and death. Surgical resection and ablation therapy have limited efficacy for patients with advanced HCC and poor liver function, so pharmacotherapy is the first-line option for those patients. Traditional antitumor drugs have the disadvantages of poor biological distribution and pharmacokinetics, poor target selectivity, high resistance, and high toxicity to nontargeted tissues. Recently, the development of nanotechnology has significantly improved drug delivery to tumor sites by changing the physical and biological characteristics of drugs and nanocarriers to improve their pharmacokinetics and biological distribution and to selectively accumulate cytotoxic agents at tumor sites. Here, we systematically review the tumor microenvironment of HCC and the recent application of nanotechnology in HCC.

**Video Abstract**.

**Supplementary Information:**

The online version contains supplementary material available at 10.1186/s12964-021-00796-x.

## Background

Hepatocellular carcinoma (HCC) is an invasive tumor that usually occurs in patients with a previous history of hepatitis or cirrhosis. Currently, HCC becomes the second leading worldwide cause of cancer-related death globally due to ascending morbidity and high malignancy [1–3]. In the past several decades, many efficient therapeutic modalities have been widely applied in HCC, such as liver transplantation, resection, tumor ablation, transcatheter arterial chemical embolization (TACE), chemotherapy, interventional radiology, biological therapy, and so on [4–7]. Early diagnosis of HCC is critical for patients and is significantly associated with patient prognosis, as interventional therapy at an early stage of HCC can greatly improve the outcome of the patients. Unfortunately, the majority of HCC patients are diagnosed in a middle or advanced stage, which lose the opportunity for curable transplantation and resection. Active invasion of blood vessels, resulting in intrahepatic and extrahepatic metastases, is attributed to a high rate of recurrence after either surgical or medical treatment, resulting in an unsatisfactory prognosis [8–11].

For patients with unresectable advanced HCC, systemic therapy is the most commonly implemented option when localized treatment and liver function are not tolerated with surgery. Targeted therapy includes sorafenib (SFB), lenvatinib, regorafenib, ramucirumab, etc., which target cancer-promoting processes at the cellular and molecular levels [12–14]. Immune checkpoint inhibitors, such as atezolizumab, nivolumab, and pembrolizumab, mainly block immune checkpoints tumors use to evade immune attack. Immune cells can massively proliferate and be activated to specifically target and annihilate tumor cells [15–17].

Despite the advances of targeted therapy and checkpoint inhibitor therapy, chemotherapy continues to remain an option for patients who have no access to these drugs due to being restricted by their economic situation [18]. These antitumor agents need to achieve and maintain adequate drug concentrations, as well as a rapid release, at the tumor site to exert efficient antitumor effects. Most antitumor drugs have obvious dose dependence; that is, increasing the drug dose can significantly improve the efficacy, but the increase in the drug dose is bound to aggravate systemic toxicity and side effects, thus limiting the clinical application of chemotherapy drugs [19, 20]. Therefore, a treatment strategy requires accurate delivery of sufficient intracellular chemicals to kill cancer cells while reducing the concentration of chemicals in nontarget organs, aiming to improve the treatment response and reduce the incidence of adverse reactions (21).

Nanotechnology, emerging as the latest approach, uses the properties and applications of materials with structural sizes ranging from 1 nm (nm) to 100 nm. Nanomaterials exhibit excellent properties in terms of optics, heat, mechanics, magnetism, and others due to their unique properties [22, 23]. Nanotechnology has demonstrated great potential for the early diagnosis and precise treatment of tumors [24].

Drug-delivering nanoparticles (NPs) are solid colloidal particles with a diameter of 10–500 nm. Drugs or active ingredients are placed inside the NPs through dissolution and encapsulation or on the surface of the NPs through adsorption and coupling. Compared to conventional drugs, NPs are designed to increase drug release in tumor sites with less drug release in normal tissues, to increase drug uptake, to protect the drugs from degradation, and to enhance drug stability [25–27]. Here, we systematically reviewed the recent progress in HCC-targeted drug delivery by nanocarriers. We want to provide a general understanding of the current state of HCC nanodrugs and propose further research into novel drug delivery nanosystems for HCC therapy.

## Microenvironment in HCC

**Fig. 1:**
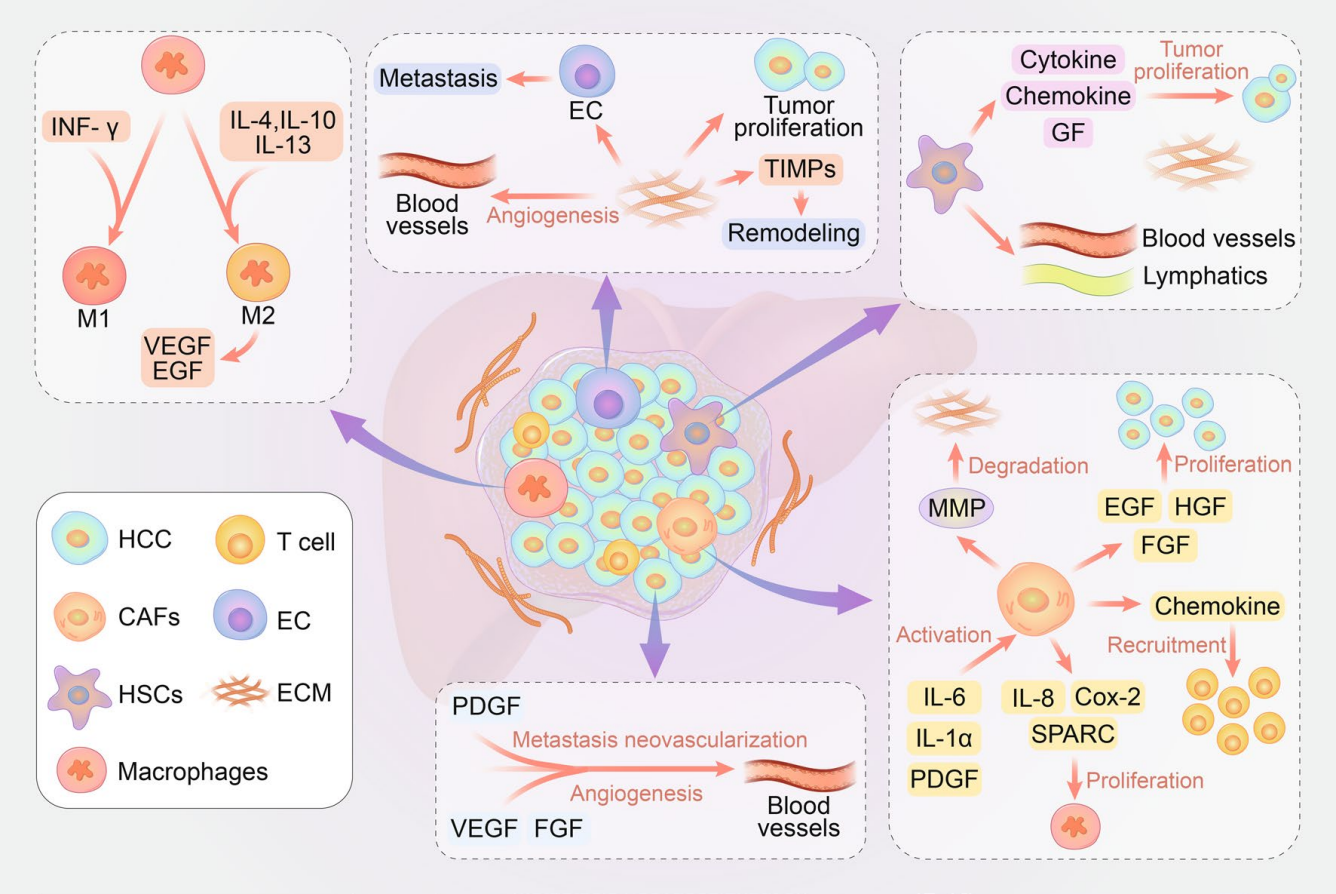
The tumor microenvironment of HCC. CAFs are activated by PDGF, IL-6, and IL-1α, which are secreted by HCC. Activated CAFs produce EGF, HGF and FGF to promote rapid proliferation; IL-8, COX-2 and SPARC to aggregate and stimulate macrophage production; MMP to degrade the ECM; chemokine to recruit T cells and maintain the protumorigenic inflammatory environment. HSCs are activated by liver inflammation, and generate cytokines, chemokines, growth factors, and ECM as well as induce the formation of tumor blood vessels and lymphatics to promote tumor growth. M1 macrophages are activated by interferon-γ, while M2 macrophages are activated by IL-4, IL-10, and IL-13. M2 macrophages produce VEGF and EGF to promote angiogenesis, growth and the invasion of tumors. Components of ECM, including collagen, elastin, hyaluronic acid, proteoglycan, polysaccharide, related enzymes, growth factors and etc., play an important role in regulating cell proliferation, promoting microenvironmental fibrosis and angiogenesis. Dysregulation of the ECM leads to cell transformation and metastasis

With the expansion of research on the tumor microenvironment (TME), we have increasingly realized that HCC might be the result of the exposure of hepatocytes to a continuously inflammatory microenvironment [28–30]. In the course of tumor progression, activated hepatic stellate cells (HSCs), cancer-associated fibroblasts (CAFs), myofibroblasts, immune cells (regulatory T cells, cytotoxic T cells, tumor-associated macrophages (TAMs), tumor-associated neutrophils (TANs), etc.) are defined as tumor stromal cells. The downregulated function of immune cells in the TME promotes the angiogenesis, progression and metastasis of tumors [31–33]. These stromal cells and the surrounding tumor stroma, consisting of extracellular matrix (ECM) proteins, growth factors, chemokines, and some stromal degrading enzymes, constitute the whole tumor [34–36]. Schematic illustration of the tumor microenvironment is shown in Fig. 1.

### Stromal cell microenvironment

The important roles of stromal cells in tumors include continuous proliferation signalling, avoidance of growth inhibition, resistance to cell death, initiation of permanent replication, angiogenesis, activation of invasion and metastasis, a reprogrammed metabolism, and evasion of immune destruction.

#### CAFs

CAFs are a major component of the tumor stroma and promote tumorigenesis, growth, and metastasis (shown in Fig. 1) [37–39]. CAFs generate collagen, fibrin, and other components of the ECM, together forming a thick matrix that supports and protects the tumor tissue, preventing drugs from reaching the tumor sites either physically or by altering signaling pathways in the physiology of tumor cells [40, 41]. In addition, CAFs contribute to immunosuppression of the TME and are an attractive therapeutic target for improving current cancer immunotherapies. CAFs are involved in the growth of HCC by producing epidermal growth factor (EGF), hepatocyte growth factor (HGF), and fibroblast growth factor (FGF), which act on adjacent cancer cells and promote rapid proliferation [42–44]. CAFs can also promote chronic inflammation by secreting chemokines to recruit T cells into tumors and maintain the protumorigenic inflammatory environment by severely suppressing the antigen-specific T cell response [45]. CAFs secrete interleukin-8 (IL-8), cyclooxygenase-2 (COX-2), and cysteine-rich acidic proteins, which aggregate and stimulate macrophage production [46–48]. HCC cells further activate CAFs by secreting soluble factors such as platelet-derived growth factor (PDGF), IL-6, and IL-1α [49].

The matrix metalloproteinase (MMP) secreted by CAFs can degrade the ECM that forms the basement membrane and promote the metastasis of tumors [50]. Therefore, the growth and metastasis of HCC depend on the interaction between CAFs and HCC cells to a certain extent. Targeting CAF-expressed molecules (e.g. extradomain splice variant of fibronectin, fibroblast-specific protein-1 (FSP-1), etc.) or secreted protumor factors (e.g., TGF-β, MMPs, vascular endothelial growth factor (VEGF), PDGF, etc.) may enhance the efficacy of immunotherapy [50, 51].

#### HSCs

HSCs infiltrate the HCC stroma, surround the tumor tissue, and localize at the tumor sinus, fibrous septum, and surrounding tumor capsule (shown in Fig. 1). The liver inflammatory response leads HSCs to be activated and transdifferentiate into myofibroblast-like cells that form the ECM and can lead to the occurrence of liver fibrosis [52, 53]. HSCs can promote the growth of HCC by generating cytokines, chemokines, growth factors, and ECM [54]. The isolation and subsequent coculture of HSCs with HCC cell lines from human tumors enhanced their proliferation, migration and expression of angiogenesis genes [55]. In addition, HSCs play an important role in the occurrence and development of HCC due to their immune suppressive function [54, 56]. The accumulation of immunosuppressive cells such as myeloid-derived suppressor cells (MDSCs) and regulatory T cells has been reported to take part in tumor immune escape [57, 58]. Activated HSCs promote the growth of HCC by inducing the formation of tumor blood vessels and lymphatics and increasing the inhibition of regulatory T cells and MDSC immunological cells in the spleen, bone marrow, and tumor tissues [59, 60].

#### TAMs

Originating from circulating monocytes, TAMs highly influence the interaction between tumor cells and stromal cells. Monocytes assemble into the TME and differentiate into TAMs through tumor-derived signals (shown in Fig. 1) [58, 61]. TAMs exist in the tumor stroma and can initiate extensive polarized activation involving stem cell niches, immunosuppression, invasion, and metastasis [61]. The activation of the TAM phenotype can be either proinflammatory via classically activated M1 macrophages or anti-inflammatory by alternatively activated M2 macrophages [62–64]. M1 macrophages are activated by cytokines such as interferon-γ, while M2 macrophages are activated by Th2 cytokines (including IL-4, IL-10, and IL-13). M1 macrophages inhibit tumor growth by generating proinflammatory and immunostimulatory cytokines, while M2 macrophages promote angiogenesis, growth and the invasion of tumors by producing either VEGF or EGF to support tissue repair and remodeling and angiogenesis [65–67]. Macrophages secrete epidermal growth factor receptor (EGFR) family ligands, including heparin-binding EGF-like growth factor (HB-EGF) and activators of signal transducer and activator of transcription 3 (STAT3), such as oncostatin M, IL-6, and IL-10 [68].

The level of TAMs is closely related to the number of cancer blood vessels in humans[67, 69]. Hypoxia is the main driving factor of tumor angiogenesis, while macrophages are recruited in areas of hypoxia in tumors, especially in necrotic tissue. Moreover, Kupffer cells are liver-specific TAMs that can generate programmed death ligand-1 (PDL-1) to interact with CD8 + T cells programmed death receptor-1 (PDR-1), decrease the cytotoxic function of CD8 + T cells, and weaken the immune-dependent response. When stimulated by inflammatory cytokines, Kupffer cells and HSCs generate a large amount of osteopontin, which involves different cellular signal transduction pathways and promotes the inflammatory response, tumor progression and metastasis [68]. Pengfei Ma et al. found that M1 macrophages significantly decreased the number of activated HSCs and enhanced the accumulation of TAMs in the fibrotic liver. M1 macrophages also increase the total and activated natural killer (NK) cells in the fibrotic liver, which can effectively attenuate liver fibrosis [70]. Oscar Yeung et al. found that the number of hepatoma cells increased 1.3–3.2 times, and more migration events were detected when hepatoma cells were co-cultivated with M2 macrophages. High M2-specific CD163 and scavenger receptor A were correlated with increased tumor nodules and venous infiltration in HCC patients [71].

### ECM

The noncellular parts of the tumor microenvironment include ECM, proteolytic enzymes (matrix metalloproteinases), growth factors, cytokines, and environmental factors [72]. The ECM is critical for maintaining the normal structure of the liver (shown in Fig. 1). An abnormal increase in surface ECM can promote the progression of HCC. In addition, cytokines and environmental factors in HCC cells and the HCC microenvironment can determine the formation of the HCC lineage. The hepatocyte microenvironment associated with programmed necrosis increases the occurrence of HCC cholangiocarcinoma, while the apoptosis-related microenvironment promotes the occurrence of HCC [73, 74].

The ECM regulates cell proliferation through a combination of components, including collagen, elastin, hyaluronic acid, proteoglycan, polysaccharide, related enzymes, and growth factors. The ECM plays an important role in supporting the liver structure and maintaining the external environment, enabling signal transduction and gene expression changes. ECM remodeling enzymes lead to microenvironmental fibrosis, characterized by increased hardness and the production of a large number of growth factors, thereby providing better mechanical support for tumor growth, while blocking the entry of chemotherapeutics, leading to drug resistance. [75, 76]. Uncontrolled collagen cross-linking and ECM sclerosis play an important role in the pathogenesis of cancer by enhancing integrin signaling, resulting in the deposition of excessive collagen type I, type II and fibronectin in the liver.

In addition, tumor cells proliferate through modulations of the integrin family. Dysregulation of the ECM directly affects epithelial cells, leading to cell transformation and metastasis [75, 77–79]. Tumor growth requires previously existing barrier rupture and hepatic tissue remodeling, mainly regulated by tissue inhibitors of MMPs. Excessive expression of MMPs can erode the basement membrane barrier and promote the invasion of cancer cells into the tissue. The ECM is also involved in endovascular lumen structure, angiogenesis, and basement membrane formation. Tumor neovascularization is more porous and permeable than normal vessels and is conducive to immune cell infiltration, metastasis, and tumor progression. The ECM can also affect immune cells, T cell activation and immune cell differentiation by blocking the normal maturation of T helper cells [73, 80, 81].

### Vascular microenvironment

HCC is a kind of hypervascularized tumor, and pathological angiogenesis is one of the most important contributors to this characteristic. During chronic liver disease, the liver damage repair response leads to fibrosis, triggering some stromal cells to secrete angiogenic factors, especially MMP, PDGF, transforming growth factor-α (TGF-α), FGF, and VEGF (shown in Fig. 1). In addition, during the process of fibrosis, changes in ECM and tissue structure will increase blood flow resistance, thus reducing oxygen exchange metabolism, resulting in hypoxia [82]. Hypoxia produces resistance to conventional therapy through multiple changes, including apoptosis, autophagy, DNA damage, mitochondrial activity, p53 and drug efflux [83].

VEGF is a very important proangiogenic factor that is expressed in degenerative nodules and gradually increases during the development of HCC [84]. Once a tumor has formed, cancer cells need a new network of vessels to provide nutrients and oxygen. Angiogenesis is a complex and tightly regulated process that is balanced by a variety of angiogenic and antiangiogenic factors between tumor and host cells. Rapid tumor growth can lead to a lack of nutrients and oxygen, stimulating the proliferation and activation of endothelial cells (ECs) [85]. ECs together with released enzymes destroy the basement membrane and eventually migrate to the final region, where they together with the ECM form vessels [86]. In addition, VEGF has a cytokine-like function that directly acts on HSCs, Kupffer cells, and hepatocytes, thus regulating the dissolution of the vascular basement membrane and interstitial matrix.

FGF, a member of the heparin-binding growth factor family, cooperates with VEGF to induce angiogenesis, while PDGF is involved in cell migration and neovascularization [87]. Cancer cells, as well as ECs and fibroblasts, secrete PDGF, which is involved in cancer progression [88]. Other important regulators of tumor angiogenesis, such as eugenin and cadherin, regulate the cellular matrix and cell-to-cell connections, thus creating conditions for the formation of new vessels [89].

### TME conditions

Hypoxia and acidic conditions are the main TME conditions due to the dramatic increase in tumorous metabolic activities and nutritional requirement. The rapid growth of hepatoma carcinoma cells requires massive amounts of oxygen, making the available oxygen insufficient, leading to hypoxia condition. Due to the limited delivery of drugs through the blood circulation, the toxic effects of chemotherapy drugs in the hypoxic area of the tumor are regrettable. In addition, increased production of reactive oxygen caused by hypoxia can interfere with DNA repair mechanisms and destabilize the genome, leading to resistance to chemotherapeutics [90]. Considering this hypoxia physiological condition, NPs are designed to be activated only when hypoxia conditions occur. The combination of NPs and bioreducible prodrugs to improve tumor hypoxic environment can enhance the therapeutic effect on tumors. On the other hand, glycolysis process is enhanced to meet the increased oxygen needs, thereby leading to the formation and accumulation of lactic acid and subsequent fermentation [91, 92]. Protons in large number are released into extracellular environment, increasing the risk of tumor metastasis and developing resistance to various anticancer drugs [93]. Similar to hypoxia condition, PH-sensitive NPs can be designed to target tumors and improve acidity, thereby reducing tumor metastasis and drug resistance [94].

A growing body of evidence suggests that the TME supports tumor progression and creates barriers to existing therapies. An increasing number of studies on the regulation of the TME to achieve better therapeutic effects have been conducted [28, 95, 96]. The tumor matrix provides hepatoma carcinoma cells with a physical scaffold for tumor growth and expansion, but also a metabolic environment (including various paracrine cytokines, chemokines, and etc.) to support tumor survival, and plays a key role in recruitment, infiltration, polarization and function of immune cells. Therefore, the exploitation of NPs to target and modify tumor matrix can enhance the effectiveness of drug treatment. NPs, by virtue of their physical and chemical properties, are able to overcome a variety of biological barriers and accumulate in tumor tissue. Based on the specific compositions and physiological conditions of TME (including hypoxia, weak acid pH and tumor pressure gradient, as well as the nature of ECM), NPs with different types of environmental stimuli responses can be designed and developed. NPs can be functionally chemically or biodecorred to accurately deliver anti-tumor drug to specific locations on the tumor. Targeting markers overexpressed on cell surface or secreted factors can increase the absorption and bioavailability of NPs by the tumor and reduce the side effects.

## NPs in vivo

Efficient drug delivery to tumor sites is a prerequisite for the high efficacy of nanodrugs. Nanodrugs use a complex five-step process to enter tumor cells in solid tumors to release drugs, namely, circulation in the blood (circulation, C), accumulation within the tumor tissue (accumulation, A), penetration diffusion in the tumor site (penetration, P), internalization by tumor cells (internalization, I), and drug release within the cells (drug release, R), collective called the CAPIR cascade conveying process [97, 98]. Before successful delivery and uptake of NPs by tumor tissue, NPs need to penetrate the vessel wall and infiltrate into the tumor tissue[99]. High cell density, dense ECM, and osmotic pressure in tumor tissues make the distribution and diffusion of nanodrugs particularly difficult. The cellular membrane is the last barrier to prevent nanodrugs from entering tumor cells. It is difficult for large nanodrugs to penetrate the cells through the membrane via diffusion but they can only be internalized into the cells through various cellular endocytosis pathways. Finally, internalized nanodrugs need to avoid lysosomal traps and degradation and to release their cargo into the cytoplasm as free drugs. When NPs are manufactured for HCC, the liver is not only a target for NPs but also one of the major obstacles to NP drug delivery. Selective delivery of therapeutic NPs to the TME is particularly at odds with rapid clearance by the liver. The interactions between HCC, NPs, and the liver become more relevant and complex.

After injection into the blood circulation, NPs nonspecifically interact with serum proteins and/or the surface deposition of antibodies and/or complement proteins, named opsonization [100]. Then, the complex will be cleared by mechanical entrapment in the pulmonary vascular bed and/or by resident macrophages of the reticuloendothelial system (RES) in the liver, spleen, and bone marrow. In this way, 60–90% of NPs are quickly cleared from blood circulation and accumulate in the liver, increasing the concentration of drugs in the liver and reducing the amount of drugs in the circulatory system, thus reducing adverse reactions to other organs. In addition, NPs need to exude through hepatic sinusoidal capillaries to reach the tumor site. Studies have shown that NPs at 20–100 nm have advantages in extravasation through fenestrated endothelium [101, 102]. The drug release mechanism of NPs includes leaching and osmosis. Diffusion through the NP skeleton (matrix), dissolution of the NP skeleton, diffusion through the polymer film, and dissolution and diffusion all occur simultaneously and are mainly controlled by both diffusion and polymer biodegradation [103].

## Targeting strategies of NPs

### Passive targeting

**Fig. 2:**
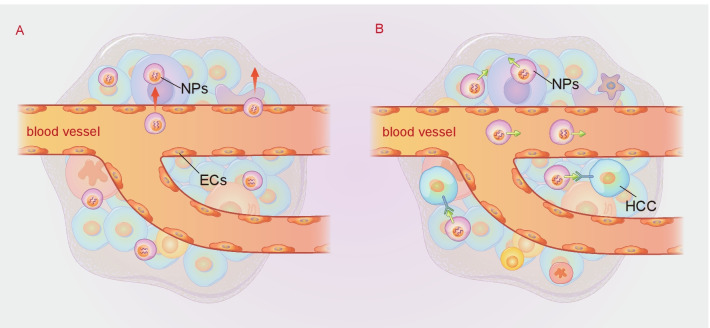
The mechanism of targeting strategies. **A** Schematic illustration of passive targeting. Enhanced vascular permeability, blood perfusion, and poor lymphatic drainage result in passive drug NP deposition and retention in tumor. **B** Schematic illustration of active targeting. Through utilizing specific ligands to bind specific complementary receptors on target cells, antitumor agents can be directed to the HCC site to exert a curative effect without damaging surrounding normal cells, tissues and organs, minimizing nonspecific uptake by untargeted cells to a great extent

Passive targeting is regarded as the accumulation of NP therapy at specific sites due to certain anatomical or pathophysiological characteristics [104]. The efficacy of passive targeting partially depends on physicochemical properties, the administration route, and the enhanced permeability and retention (EPR) effect, among which EPR has the most important impact [104–106]. In passive targeting, the intrinsic properties of tumors, such as enhanced vascular permeability and poor lymphatic drainage, greatly promote the EPR effect, resulting in more NP deposition in tumor tissues (shown in Fig. 2) [106, 107]. Normal tissues have microvascular endothelial gap density and a complete structure, so macromolecular and lipid particles cannot easily pass through the blood vessel walls. Conversely, macromolecular substances and lipid particles in solid tumor tissues have selective high permeability and deposition in the TME for a long time due to 1) euangiotic blood vessels, 2) wide vascular wall space, 3) poor structural integrity, and 4) lack of lymphatic reflux [29, 32]. The EPR effect is thought to be a size-dependent phenomenon, and only macromolecules greater than 5 nm can escape renal clearance and target tumors by the EPR effect. Rapid renal filtration significantly reduces the blood concentration of NPs, resulting in rapid removal of NPs from the tumor and decreasing targeting efficiency [108]. In addition, few small molecules remain in tumors because they are easily pushed back into the bloodstream. Therefore, NPs should be designed to effectively prolong their blood circulation by reducing renal excretion and phagocytosis of RES. NP therapeutics enhance passive accumulation of antitumor agents in the liver and improve therapeutic effects [109]. This effect also helps in the accumulation of higher molecular weight compounds inside the tumor [110, 111]. Furthermore, tumor regression and longer survival than conventional agents were observed when therapeutic NPs were used, indicating the potency of the antitumor effect of NPs [112, 113]. Conventional inorganic NPs could escape the rapid filtration of the kidney, while NPs with polyethylene glycol (PEG)-coated surfaces could slow down the absorption of RES [114, 115]. As a result, they have high tumor-targeting efficiency due to the EPR effect, with longer and higher blood retention times and concentrations.

### Active targeting strategy

Active targeting can increase the specificity of drug delivery against cancer by functionalizing NPs by utilizing markers or ligands to bind specific complementary receptors on target cells (shown in Fig. 2) [116]. Through the structural modification of NPs and the connection of specific ligands, antitumor agents can be directed to the HCC site to exert a curative effect without damaging surrounding normal cells, tissues and organs, minimizing nonspecific uptake by untargeted cells to a great extent [117, 118]. Active targeting strategies mainly depend on the specificity and affinity of the selected ligands to cancer cells. Researchers have used proteins (antibodies and transferrin), peptides, nucleic acids, small molecules (anisamide, folate, etc.), aptamers, and polysaccharides as ligands that preferentially bind to tumor cells [119]. In the field of active targeting therapy for HCC, the active targeting effect of immune-targeted agents and magnetic NPs has been widely reported.

Glypican-3 (GPC3) is a heparan sulfate proteoglycan that is overexpressed in both the cell membrane and cytoplasm in HCC. GPC3 actively regulates HCC tumor growth, and its expression is related to a poor clinical prognosis of HCC. GPC3 has been validated as an effective immunotherapeutic target for HCC in vitro and in vivo [120–122]. In the HepG2 cell line, SFB-loaded polymer NPs modified with an anti-GPC3 antibody (NP-SFB-Ab) exhibited higher cellular uptake, better stability, a higher concentration of SFB in the cell culture medium, and higher cytotoxicity to hepatoma cells. In addition, NP-SFB-Ab significantly inhibited the growth of HepG2 xenograft in nude mice [123]. In phase I trials, chimeric antigen receptor (CAR)- GPC3 T-cell therapy exhibited initial safety and efficacy in inhibiting HCC [124]. Rui Tian et al. constructed a GPC-3-targeted hybrid Fe3O4 core/Au shell nanocomplex (FANP) that exhibited effective tumor ablation by photothermal therapy with minimal toxicity and side effects [125].

Asialoglycoprotein receptor (ASGPR), exclusively expressed on hepatocytes, specifically recognizes and binds to galactose (GAL) and galactosamine (GALNAC) residues [126]. However, the simultaneous expression of galactose receptors on both normal hepatocytes and hepatoma cells may limit their clinical application to a certain extent due to potential off-target effects. ASGPR was reported to be overexpressed in some liver diseases, such as acute and chronic viral hepatitis and autoimmune hepatitis. Therefore, for HCC that contributes to viral hepatitis or autoimmune hepatitis, a single ASGPR-targeting strategy is likely toxic to surrounding liver tissue [127]. Dual-targeting ligands for more precise targeting therapy have been studied in vivo and in vitro experiments. In Ya Xiang’s study, a dual-targeting strategy using folic acid (FA) and galactosylated chitosan-5-fluorouracil acetic acid as ligands demonstrated higher efficacy in specific targeting in vitro and in vivo compared to a single targeting strategy [128].

CD44, a highly distributed cell surface transmembrane glycoprotein, mediates cell–cell and cell–matrix interactions and is implicated in cell adhesion, tumor growth and metastasis. Heterogeneous adhesion plays a promoting role in tumor cell invasion and metastasis. Hyaluronic acid (HA), a widely distributed naturally biodegradable and biocompatible linear polysaccharide, can bind specifically to CD44. Studies have demonstrated that HA could promote the efficacy of drug delivery [129–131].

The arginine-glycine-asparagine (RGD) tripeptide is an important component of cellular interactions, specifically targeting integrins. Integrins are heterodimer transmembrane glycoproteins that regulate cell adhesion, migration/invasion, proliferation, survival, and apoptosis. RGD has a high affinity for integrin and inhibits the interaction of ECM proteins with integrin. Due to their functions in cancer biology and the availability of small molecule ligands, RGD-bound integrins have been identified as attractive in vivo targets for tumor molecular imaging [132–134].

The rapid proliferation of tumor cells requires more organic compounds and important nutrients, including vitamins such as FA, biotin, retinoic acid (RA), and dehydroascorbic acid (DHAA) [135]. Folic acid is a small molecule used to actively target cancer cells. There are two main types of folic acid receptors: normal cells express a reductive folic acid vector with low affinity, which can only transport the reduced form of folic acid, while cancer cells express a folic acid receptor with high affinity, glycolipinositol-linked folic acid receptor, which can transport the two folic acid- and folate-linked NPs. The advantages of folic acid as a targeting agent include its non-immunogenicity and specificity [136–139]. In addition, transferrin is a serum glycoprotein that transfers iron from the blood to cells by binding to transferrin receptors on cell membranes. These receptors are highly expressed in metastatic and drug-resistant cancer cells [139, 140].

### Stimulus-responsive release at the target site

Changes in pH, redox potential, hypoxia, hyperthermia, expression of certain enzymes, proteins, and stromal cell content in the TME are different from those in other tissues [29, 32]. These differences are used to release the drugs from the NPs. Similarly, external stimuli (such as heat, ultrasound, light, and magnetic fields) can be used to target the tumor [141]. Light can be used in photothermal, photodynamic, and photoacoustic applications because specific nanomaterials are characterized by the ability to absorb light of various wavelengths. After light treatment, light energy is converted to heat or electrons, and the properties of nanomaterials, such as temperature, surface hydrophobicity, morphology, or chemical reactivity, can be changed. Therefore, many researchers use light to control the particle size to enhance drug distribution.

Ultrasound-responsive NPs can be used for tumor therapy or imaging. With remarkable advances in ultrasound technology, ultrasound has gained widespread attention as a powerful method of drug delivery because it can make NPs go deep into organs and trigger gas production, allowing drugs to be released at certain sites [142].

Magnetic NPs, such as superparamagnetic iron oxide NPs (SPIONs), have been developed for use in hyperthermia, drug delivery, and image guidance [143]. When an alternating magnetic field is applied, the magnetic NPs vibrate in accordance with the direction of the magnetic field, which increases the ambient temperature [144]. Therefore, some researchers have used this property to improve tumor permeability in combination with hyperthermia or drug delivery. Magnetic NPs are easily made into evenly dispersed magnetic fluids, which is convenient for drug administration. After being ingested by tumor cells, the magnetic NPs can be evenly dispersed in the tumor and can enter daughter cells with cell division to exert a killing effect. Currently, stimulus-responsive release combined with immunotherapy has shown great potential in inhibiting metastasis and recurrence of HCC [144, 145].

### Liver targeted gene therapy

Gene therapy is applied to treat diseases by transferring therapeutic nucleic acids (i.e., plasmid DNA, siRNA, or microRNA) to introduce new genes or restore, increase, or stop gene expression (142). The introduction of target genes into tumor cells is the basis for the expression of exogenous genes to exert biological effects, and the search for safe and effective gene carriers is an important part of gene research. NPs can carry a variety of genes, not only increasing the numbers of the target gene in the cells but also enhancing their ability to resist nuclease damage and prevent degradation. The main limitations are the lack of targeted gene therapy and the efficiency, safety and capacity of gene transfer carriers [146], challenges that remain to be solved [30, 147].

The advantage of using nanocarriers to transport nucleotides is that they can target and transport nucleotides, reduce the killing effect on normal cells and avoid systemic toxicity. Given the multiplicity of hyperactive immunosuppressive forces acting within the HCC microenvironment, combined therapy might be an option [148, 149].

## Types of NPs

Currently, NPs widely studied for specifically targeting HCC include organic NPs (bionanocapsules), inorganic NPs (magnetic and metallic NPs, carbon structure, etc.), lipids, and polymers. Table 1 demonstrates the reported clinical trials investigating the use of nanostructures whose endpoint is the treatment of hepatocellular carcinoma. Common types are listed as follows:

**Table 1:** Clinical trials investigating nanostructures whose endpoint is the treatment of hepatocellular carcinoma

Clinical trial name	Phase	NP type	Loaded drug	NP target	Trial number
ThermoDox study	3	Thermally sensitive liposome	Doxorubicin	Stimulus-responsive release	NCT00617981
ThermoDox study	3	Thermally sensitive liposome	Doxorubicin	Stimulus-responsive release	NCT02112656
DCR-MYC study	1b/2	Lipid nanoparticle	siRNA Oligonucleotide	Liver targeted gene therapy	NCT02314052
Nano Drug Interventional Therapy	1/2	Glycyrrhizin mix with gemcitabine	Gemcitabine	Active targeting	NCT02449109
PLM60 study	1	Liposome	Mitoxantrone hydrochloride	Passive targeting	NCT04331743
OPTIMA study	3	Thermally sensitive liposome	Doxorubicin	Stimulus-responsive release	NCT02112656
ThermoDox study	1	Thermally sensitive liposome	Doxorubicin	Stimulus-responsive release	NCT00441376
OUTREACH study	1	Liposome	Double stranded RNA	Liver targeted gene therapy	NCT02716012
L-NDDP study	1/2	Liposome	Aroplatin	Passive targeting	NCT00057395
TLC D-99 study	2	Pegylated liposome	Doxorubicin	Passive targeting	NCT00003296
NIFE study	2	Liposome	Irinotecan	Passive targeting	NCT03044587
ReLive study	3	Water insoluble polymer	Doxorubicin	Passive targeting	NCT01655693
MRX34 study	1	Liposomal mimic	MicroRNA-34a	Liver targeted gene therapy	NCT01829971

### Liposomes

Liposomes are spherical vesicles with a hydrophilic cavity surrounded by one or several lipid bilayers that allow the encapsulation of drugs with different solubilities [150]. Liposome agents can specifically target tumors by binding to antibodies such as gender-affirming hormone (GAH), anti-EGFR, or anti-human epidermal growth factor receptor 2 (HER2) monoclonal antibodies, small molecules such as folic acid and transferrin, or tumor-targeting peptides such as RGD rings [151–153]. Nanoliposomes with a particle size of approximately 100 nm, after surface modification with hydrophilic materials such as polyethylene glycol, have the characteristics of "long circulation", "stealthy" and "stereostable", which can reduce the phagocytosis of drugs by liver macrophages, improve drug targeting, hinder the binding of blood protein components and phospholipids, and extend the circulation time [154]. Nanoliposomes can also improve the oral absorption of biomolecular drugs and their absorption via other drug delivery routes, such as transdermal insulin liposomes [155]. In addition, pegylated cationic liposomes are commonly used for the loading and delivery of siRNA and can improve the stability of siRNA [156].

As a drug carrier, liposomes have the following advantages: (1) they are mainly comprised of natural phospholipids and cholesterol, which will be biodegraded and will not accumulate in the body, with low immunogenicity; (2) both water-soluble and lipid-soluble drugs can be embedded for delivery, and the drug is slowly released from the liposome for a long time; and (3) through endocytosis and fusion, liposomes can directly deliver drugs into cells, avoiding the use of high concentrations of free drugs and thus reducing adverse reactions (157–160). Liposomes are characterized by biocompatible, flexible formulations, the potential to add targeting moieties, etc. However, there are still some limitations, such as being a poor universal carrier, invariant size and shape, and poor drug release characteristics.

### Polymers

Polymers are large molecules made up of repeated subunits called monomers, and polymer NPs consist of macromolecular materials encapsulated or attached to a surfactant. Several natural and synthetic polymers have been used to manufacture NPs for drug delivery, including polysaccharides, proteins, amino acids, poly(ethyleneimine), poly(cyanoacrylate), poly(methylene malonate), and polyesters [161–163]. Haipeng Wang et al. modified sorafenib-loaded BSA NPs (SRF-BSANPs) with folic acid to obtain FA-SRF-BSANPs, which can promote the intracellular uptake and tumor targeting of hepatoma cells (SMMC-7721) with the strongest inhibitory effect compared with SRF-BSANPs and sorafenib solution [164]. Similar to liposomes, many polymer-based NPs are easy to manufacture and have good biocompatibility and biodegradability. However, polymer NPs have limited stability and dose-dependent toxicity in vivo, an invariant size and shape, and poor drug release characteristics [163, 165].

### Metallic and magnetic nanocarriers

Inorganic NPs applied for diagnosis and treatment include superparamagnetic NPs (ironoxide NPs), quantum dots, and plasmonic NPs (gold and silver NPs) [166]. Magnetic NPs (MNPs), most commonly iron oxide NPs, use magnets to deliver therapeutic agents precisely to the target area. Magnetic Fe3O4 NPs have shown promise as drug carriers for treating lung and liver tumors in vivo [167]. Gold NPs (AuNPs) are characterized by a high specific surface to area volume ratio, stable properties, multifunctional properties, easy synthesis, high permeability and retention effects, and photothermal conversion capability [168]. Under near-infrared irradiation, the nanocarrier can rapidly convert near-infrared light into heat, increase cell absorption, and trigger drug release [168, 169]. Huixiang Ju et al. explored the potential therapeutic effects, biomechanics, and toxicity of a combination of MNPs and extremely low-frequency electromagnetic field (ELFF) exposure in vitro. Flow cytometry for anti- alpha fetal protein (AFP) antibody, which coated the MNPs, indicated that the combined treatment induced Bel-7402 and HepG2 hepatoma cell lines to undergo apoptosis without significant effects on healthy hepatic cells [170]. However, the nonbiodegradable nature of magnetic and metal NPs in tissues leads to the accumulation of NPs, which limits repeated applications. As imaging agents or photothermic therapies, AuNPs can accumulate for months after injection, particularly in the liver and spleen, but AuNPs have no obvious toxicity [168].

## Side effects/toxicity of NPs in cancer therapy

Nanomaterials can be used for targeted therapy at specific sites of disease, which helps to reduce the off-target toxicity of many drugs. However, nanocarriers sometimes produce nanotoxicity due to potential off-target effects or activate autoimmunity and cause subsequent attacks that can lead to adverse effect [171].. NPs can reach areas that large particles cannot reach due to their small size and high stability. NPs may easily enter organelle such as mitochondria, endoplasmic meshes, lysosomes and nuclei through pores in biofilms, and catalyze chemical reactions with biomolecules, altering the normal three-dimensional structure of biomolecules and biofilms [172, 173]. In this way, NPs may lead to inactivation of some hormones and important enzyme systems in the body [174]. NPs can cause oxidative stress, inflammatory reactions, DNA damage, apoptosis, cell cycle changes, abnormal gene expression, and damage to the lungs, cardiovascular system and other tissue organs [175]. Currently marketed nanomedicines will still be ingested by the RES and stored in normal tissues such as the liver and spleen, resulting in toxicity. The destruction of nanostructures, which have electronic, optical, and magnetic properties, can lead to unpredictable and unique toxic effects [176]. Nanostructures can be distributed as complete NPs to multiple organs or metabolized into multiple fragments, which allow them to enter different organs and accumulate for some time before being excreted.

## Conclusions

The rise and development of nanotechnology bring new hope for conquering malignant tumors. Drug delivery systems in the targeted therapy of liver cancer have made enormous advances, and they have shown good efficacy in animal models and human trials, indicating their broad application prospects in the treatment of cancer. Some NPs have shown good liver targeting, slow release, and modifiability, which can increase the concentration of drugs in the liver, enhance treatment efficacy and reduce the toxicity and side effects of drugs.

In addition to causing tumor progression (including invasion and metastasis), abnormalities in TME can also lead to pharmacologically dynamic (related to drug delivery) and pharmacological dynamic (sensitivity-related) resistance. Abnormal components in TME, such as abnormal tumor vascular system, deregulation of ECM, interstitial hypertension (elevated interstitial fluid pressure), etc., co-prevent the distribution of drugs. The goal of nanotherapy is that patients can be selected strictly based on their characteristics of tumor complexity and heterogeneity; therefore, they can benefit from nanotherapy to the maximum extent and achieve the purpose of precision therapy. The antitumor nanodrugs may target a gene or a molecule due to specific modifications and optimization or be designed according to the results of individual gene sequencing to form personalized and specific drug therapy, which is the trend of the future treatment of tumors. It is believed that with the continuous innovation and progress of nanotechnology, the selection of materials and the preparation process will continue to be optimized, the biological safety of nanomaterials will be improved, the metabolism and distribution of nanomaterials in the human body will become controllable, and the biocompatibility will be further improved. New nanodrugs and gene carriers with excellent performance will certainly emerge, providing a new way to cure refractory diseases such as tumors.

## Data Availability

Not applicable.

## Abbreviations

HCC: Hepatocellular carcinoma
TACE: Transcatheter arterial chemical embolization
nm: Nanometer
NP: Nanoparticle
TME: Tumor microenvironment
HSC: Hepatic stellate cell
CAF: Cancer-associated fibroblast
TAM: Tumor-associated macrophage
TAN: Tumor-associated neutrophil
ECM: Extracellular matrix
EGF: Epidermal growth factor
HGF: Hepatocyte growth factor
FGF: Fibroblast growth factor
IL: Interleukin
COX-2: Cyclooxygenase-2
PDGF: Platelet-derived growth factor
MMP: Matrix metalloproteinase
FSP-1: Fibroblast-specific protein-1
VEGF: Vascular endothelial growth factor
MDSC: Myeloid-derived suppressor cell
EGFR: Epidermal growth factor receptor
HB-EGF: Heparin-binding EGF-like growth factor
STAT3: Signal transducer and activator of transcription 3
PDL-1: Programmed death ligand-1
PDR-1: Programmed death receptor-1
NK cell: Natural killer cell
TGF-α: Transforming growth factor-α
EC: Endothelial cell
VEGFR: Vascular endothelial growth factor receptor
FGFR: Fibroblast growth factor receptor
RES: Reticuloendothelial system
EPR: Enhanced permeability and retention
PEG: Polyethylene glycol
GPC3: Glypican-3
SFB: Sorafenib
CAR: Chimeric antigen receptor
ASGPR: Asialoglycoprotein receptor
GAL: Galactose
GALNAC: Galactosamine
FA: Folic acid
HA: Hyaluronic acid
RGD: Arginine-glycine-asparagine
RA: Retinoic acid
DHAA: Dehydroascorbic acid
SPION: Superparamagnetic iron oxide NP
GAH: Gender-affirming hormone
HER2: Human epidermal growth factor receptor 2
AuNP: Gold NP
MNP: Magnetic NP
ELFF: Extremely low-frequency electromagnetic field
AFP: Alpha fetal protein
